# The Methyl Functionality of Monolithic Silica Xerogels Synthesized via the Co-Gelation Approach Combined with Surface Silylation

**DOI:** 10.3390/gels9010033

**Published:** 2022-12-30

**Authors:** Selay Sert Çok, Fatoş Koç, Zoltán Dudás, Nilay Gizli

**Affiliations:** 1 Chemical Engineering Department, Ege University, İzmir 35100, Turkey; 2 Centre for Energy Research, Neutron Spectroscopy Department, 1121 Budapest, Hungary

**Keywords:** xerogels, silylation, co-gelation

## Abstract

The present research aims to investigate the chemical and morphological properties of the methylated silica xerogels produced via the co-gelation approach combined with surface silylation. In the sol–gel synthesis, methyltrimethoxysilane (MTMS) and tetraethylorthosilicate (TEOS) were utilized as silica precursors and trimethylchlorosilane (TMCS) served as a silylating agent. Structural changes were observed depending on the MTMS/TEOS molar ratio and on the post-synthesis-performed surface silylation of the xerogels. Post-synthesis silylation plays a critical role in the exchanging of the surface silanols with methyl groups, preserving the monolithic form. The morphological and structural changes were followed by SEM, ^29^Si-MAS-NMR, FTIR spectroscopy, nitrogen porosimetry, and contact angle measurements. The results have shown significant structural variations depending especially on the MTMS content. With an increasing MTMS content, the morphology of the samples has changed from a micro/mesoporous texture to a meso/macroporous texture. A higher degree of methyl substitution has been achieved for the silylated samples both confirmed by the FTIR and ^29^Si-NMR results. On the other hand, only the samples with a high MTMS content could preserve their structural integrity after evaporative drying, and all have exhibited a high degree of hydrophobicity with θ > 140°.

## 1. Introduction

Silica-based xerogels are sol –gel-derived porous materials synthesized in ambient conditions. Depending on their morphology, they can combine many exceptional properties such as a high apparent surface area, low density, high porosity, and low thermal conductivity; therefore, they have been proven to be promising materials in diverse applications such as sorption of organic liquids/oils [1,2], thermal insulation [3,4,5], water filtration [6], biotechnological applications such as implantable devices [7,8], scaffolds [9], and drug carrier systems [10,11]. However, their moisture sensitivity is one of the major obstacles hindering their practical application. Fortunately, silica-based xerogels have a tunable surface chemistry, and their hygroscopic properties can easily be tailored depending on the target application.

Basically, the choice of the silica precursor used in xerogel synthesis has a crucial impact on the chemical and structural properties and on the surface chemistry of the final material. For instance, silica xerogels prepared with conventional tetraalkoxysilanes such as tetraethylorthosilicate (TEOS) and tetramethylorthosilicate (TMOS) are rich in surface silanol groups (Si-OH), which are the main source of hydrophilicity. These silanol groups promote condensation reactions during solvent evaporation, ultimately resulting in an extensive irreversible shrinkage [12]. The irreversible shrinkage can hinder the material from preserving its porous form and structural integrity during evaporative drying. As an effective solution, Si-OH polar groups can be replaced with hydrolytically stable Si-R (R = alkyl or aryl) groups that inhibit the material wettability and change the nature of the aerogel from hydrophilic to hydrophobic [13]. The hydrophobization reaction not only prevents material degradation caused by moisture over time, but also promotes the spring-back effect during drying and protects the porous monolithic structure against network collapse or shrinkage [3,14,15,16].

Hydrophobic silica aerogels can be obtained either by performing silylation before gelation or by using the co-precursor method. In both strategies, organically modified silica precursors (i.e., alkylsilanes or arylsilanes of type R_4-x_Si(OR’)_x_) serve as the chemical modifiers of the silica particles derived from the silicon alkoxide (Si(OR’)_4_) precursor. The apparent surface free energy of silica xerogels can be reduced using these organofunctional silanes, which replace the surface polar –OH groups with non-polar –CH_3_ groups [17]. In both approaches, the attachment of radical methyl groups to the surface of silicon alkoxide-based particles leads to a differentiation in the hygroscopic behavior of the material and renders the structure hydrophobic. Moreover, by adjusting the organofunctional silane/silicon alkoxide molar ratio and the synthesis pH, it is easy to change the nature of the xerogel from hydrophilic to hydrophobic [11,18,19].

Although both techniques are commonly accepted and widely used, each has advantages and disadvantages. For example, although silylation is an effective method for achieving high hydrophobicity, it is a slightly time-consuming strategy because it generally requires tedious post-modification steps [20]. The co-gelation approach, on the other hand, is a more cost-effective and time-saving strategy. It significantly lowers the need for an extra solvent as it eliminates the need for modification after gelation. Due to the existence of surface radicals introduced by the trifunctional alkoxysilanes in this approach, the inter-chain bonding of the xerogels during sol–gel polymerization is also reduced, and this results in a looser solid matrix. As the network gets looser, the severe capillary tension that normally arises during solvent evaporation can be eliminated, and the monolithic structure can also be retained well using this approach.

Silylation is simply about the end-capping of the reactive silanol that remains after gelation, and by modifying the wet gel surface with an effective monofunctional silylating agent like trimethylchlorosilane (TMCS) these open silanols can effectively be covered with methyl radicals [21,22,23]. On the other hand, in the co-gelation approach, during the condensation of tri-functional silanes like methyltrimethoxysilane (MTMS), in addition to the inclusion of one non-polar methyl group to the system during gelation, three methoxy groups ready to undergo polymerization reactions are also incorporated. Unless a complete condensation of these polar alkoxide groups occurs in the network, free silanol polar groups will remain in the network after the drying step. This makes the performing of an additional silylation after gelation helpful in enhancing the methylation of the silica xerogels derived by the co-gelation approach.

In the studies focusing on the synthesis of MTMS-derived aero/xerogels in the literature, MTMS is incorporated in sol–gel reactions as a trifunctional silica precursor that can impart the material’s inherent hydrophobicity and improve the mechanical strength [16,24,25,26]. In most of these studies, the methylation of the samples has been accomplished only by polycondensation of MTMS (the in-situ approach) eliminating all post-gelation modification steps [26,27,28,29,30,31]. In addition to this approach, there are other studies in which the in-situ modified MTMS-derived aero/xerogels were subjected to additional post-modification steps (usually silylation with monofunctional silanes) to further improve their hydrophobicity or mechanical durability [32,33,34,35]. Applying additional silylation can pose a challenge, especially for a large-scale production since many solvents are being used during the washing steps after silylation, additional chemicals are required and the required time for the synthesis can be extended. On the other hand, including silylation as a post-modification step and combining these two approaches may create a synergistic impact and can significantly enhance the xerogel’s surface and microstructural properties. Until recently, the application of additional silylation in MTMS-derived aero/xerogels has become the individual preference of the researchers. The existing studies that combine both in-situ and ex-situ silylation for an effective methyl substitution to the xerogel network are very limited [36,37]; therefore, the possible synergetic effect of combining these two approaches is still poorly understood and needs to be systematically explored.

The present study aims to investigate the surface and pore properties of the methylated silica xerogels produced via the co-gelation approach or in combination with surface silylation. In the present study, a trifunctional organosilane and methyltrimethoxysilane were utilized as the silica co-precursors along with the conventionally used tetraethylorthosilicate. In order to observe the structural changes in the materials with methyl substitution, the molar ratio of MTMS/TEOS was varied, and an extra silylation with a monofunctional silane (trimethylchlorosilane) was also investigated as a second parameter. Various analyses, namely, scanning electron microscopy (SEM), nuclear magnetic resonance spectroscopy (^29^Si-MAS-NMR), Fourier-transform infrared spectroscopy (FTIR), N_2_ sorption, and contact angle measurements were performed to chemically and morphologically characterize the obtained xerogels.

## 2. Results and Discussion

### 2.1. Chemical Characterization

#### 2.1.1. FTIR Spectroscopy

**Figure 1:**
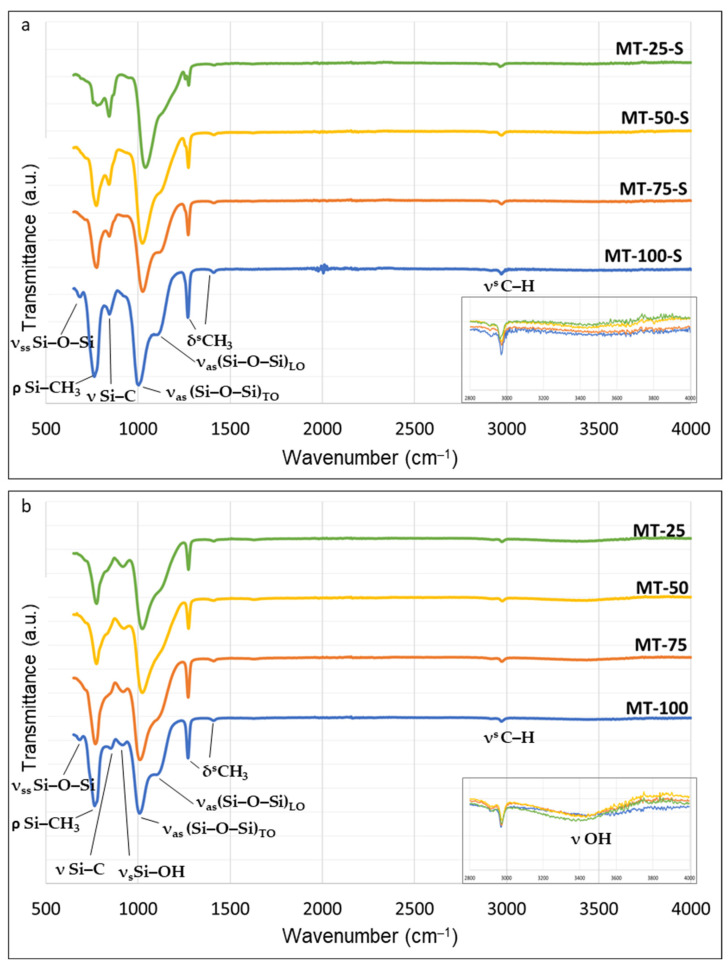
The FTIR spectrum of the (**a**) silylated and (**b**) non-silylated silica xerogels.

The FTIR spectra of the synthesized xerogels given in Figure 1 revealed various bands corresponding to the different structural units of the silica network. These bands were assigned to different characteristic vibrations. The band intensity qualitatively showed us the related chemical groups existing in the obtained xerogel network.

In Figure 1, the intense silicon–oxygen covalent bonds that appeared in the 1000–1150 cm^−1^ range were related to the asymmetrical stretching vibration of the siloxane bonds confirming a three-dimensional (3-D) silica network. According to the spectra, all samples exhibited a high degree of polycondensation regarding intense Si–O–Si peaks in this range and it was in a good accordance with the NMR data. For the samples synthesized with 100% MTMS (i.e., MT-100 and MT-100-S), the additional peaks observed at 680 cm^−1^ as shoulders were also related to the symmetrical stretching vibration of Si–O–Si bond in the solid skeleton [38].

Figure 1 also clearly depicts that, when the amount of MTMS in the system increased, different vibrational Si–O–Si structures between 1000–1200 cm^−1^ appeared as new shoulders. Consequently, the broad band obtained at approximately 1075 cm^−1^ became narrower and more intense. The depth of these shoulders increased with the MTMS content. These emerging peaks were attributed to the various structures forming the siloxane bonds resulting from the two optical modes of the ν_as_ Si–O–Si vibration in the spectra caused by the Coulomb interactions, namely, the transverse mode (TO) (1100 and 1000 cm^−1^) and the longitudinal mode (LO) (1250 and 1100 cm^−1^) [39,40]. Evidently from Figure 1, the increasing amount of trifunctional organosilane as the precursor, favored the formation of new siloxane structures with the LO_4_-TO_4_ vibrational modes (ν_as_ (Si–O–Si)_TO_, at ~1075 cm^−1^ and ν_as_(Si–O–Si)_LO_ at ~1180 cm^−1^) in the silica matrix [18]. These additional formations ultimately caused an increase in the solid skeleton density and the network compaction and decreased the porous morphology as proven by the SEM images and the N_2_ porosimetry results in the subsequent sections.

Depending on the methyl attachment to the silica xerogel surface, distinct peaks corresponding to the different vibration modes of the Si–CH_3_ bonds appeared in the spectra. For example, the strong signals obtained at around 770 cm^−1^ can be attributed to the asymmetrical stretching vibration of the Si–C bonds and the intensity of the signals increased correspondingly with the methyl content in the samples. The signals appearing as shoulders near 840 cm^−1^ were related to the stretching vibrations of the Si–C and were indeterminate in the unmodified samples compared to the silylated ones [41]. The peaks obtained at 1270 cm^−1^ corresponded to the asymmetric deformation of the C−H bonds and were visible in all samples. The varying intensity of this signal was also directly related to the amount of methyl substitution in the silica network and is at its highest in the sample synthesized via surface silylation with 100% MTMS content (MT-100-S). The slight signals appearing at around 1410 cm^−1^ and 2970 cm^−1^ were attributed to the asymmetric deformation (bending) of the C–H bonds and the symmetrical stretching of the C–H bonds, respectively, and were visible in all samples. The non-silylated silica samples depicted a large band centered at around 3500 cm^−1^, which can be attributed to the different –OH group vibrations [41]. This band was present in the MT-25, MT-50, and MT-75 samples. The band intensity decreased with the increasing quantity of functionalization, highlighting an increase in hydrophobicity (the decreasing of the number of –OH groups).

As a critical observation, the absence of any obvious—OH related signals at the wavelengths 3500 cm^−1^ (ν OH) and 960 cm^−1^ (ν_s._ Si–OH) at the spectra recorded from the silylated samples proved that the polar hydroxyl groups were successfully replaced by non-polar radical groups and a hydrophobic structure was achieved in all samples.

#### 2.1.2. ^29^Si-MAS-NMR

In the silicon spectra, the signals that appeared between −70 to −120 ppm were assigned to the existence of a ^29^Si nucleus in a tetrahedral oxygen environment, which is usually shown by a Q^n^ notation. In short, a Si atom was coordinated by n-bridging oxygen atoms (BO) and (4-n) non-bridging oxygen atoms (NBO) [2,42,43]. The broad spectrum of the ^29^Si chemical shifts is also separated into five specific regions in terms of various structural units: isolated orthosilicate structures (Q^0^, −66 to −74 ppm), linked tetrahedra in pyrosilicates (Q^1^, −77 to −82 ppm), geminal silanols (Q^2^, −85 to −89 ppm), free silanols (Q^3^, −92 to −100 ppm), and tetrahedra 3D cross-linked networks (siloxanes) (Q^4^, −103 to −115 ppm) [44].

**Figure 2:**
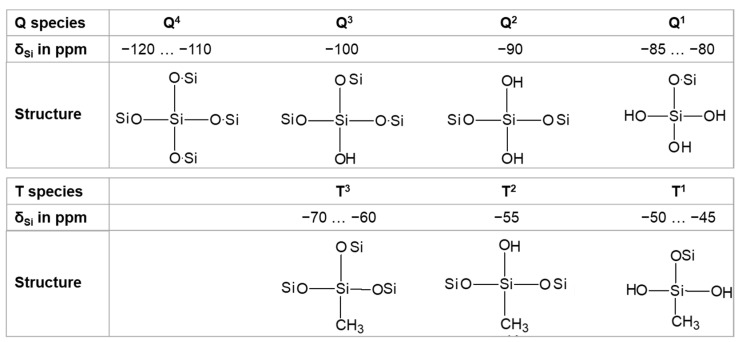
Structures and signals of silica and trifunctional silanes in the ^29^Si-MAS- NMR spectrum.

The T^n^ resonances resulting from the condensation of functional silanes (i.e., Si atoms in the trifunctional alkoxysilane in the present study) were also observed in the spectra of functionalized silica and classified into three ranges in which the Si atoms were connected either with one carbon neighbor and three BOs (T^3^), or two BOs and one NBO (T^2^), or one BO and two NBOs (T^1^) [45]. The connection between the trialkoxysilane functional groups and the surface silanols indicated a possibility for the formation of three different types of anchoring structures on the silica particles. These anchoring structures are referred to as mono- (T^1^), bi- (T^2^), and tridentate (T^3^) structures in terms of the reaction of one, two, or three alkoxy groups of MTMS between the surface silanols. T^3^ was related to the fully condensed Si species (CH_3_Si (OSi)_3_ at the band near −67 ppm, whereas T^2^ and T^1^ were related to the Si species with either one or two siloxane bonds at signals near −57 and −50 ppm, respectively. Figure 2 depicts the related chemical shifts and their commonly assigned structures.

**Figure 3:**
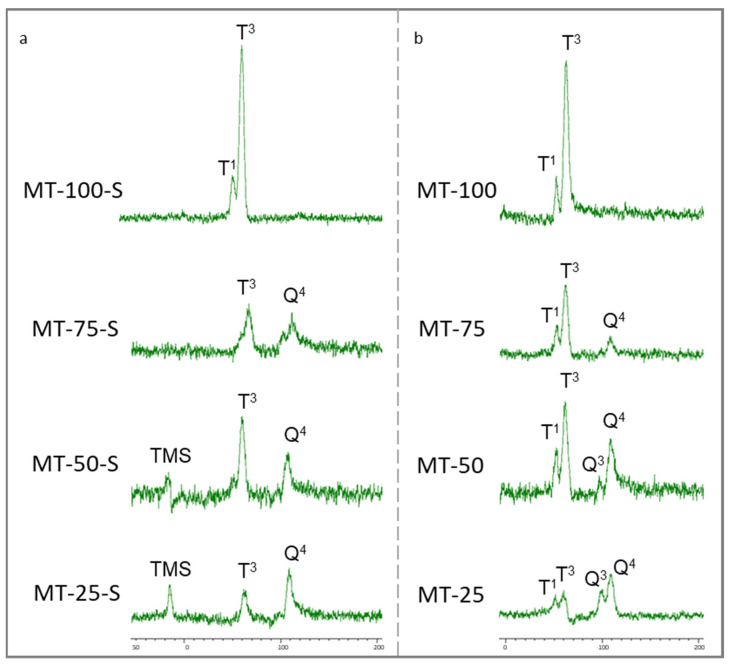
^29^Si MAS NMR spectra of the (**a**) silylated and (**b**) non-silylated silica xerogels.

Figure 3 illustrates the 29Si-MAS-NMR spectra for the silica xerogels, which helped identify the molecular-level structural changes depending on the methyl content.

**Figure 4:**
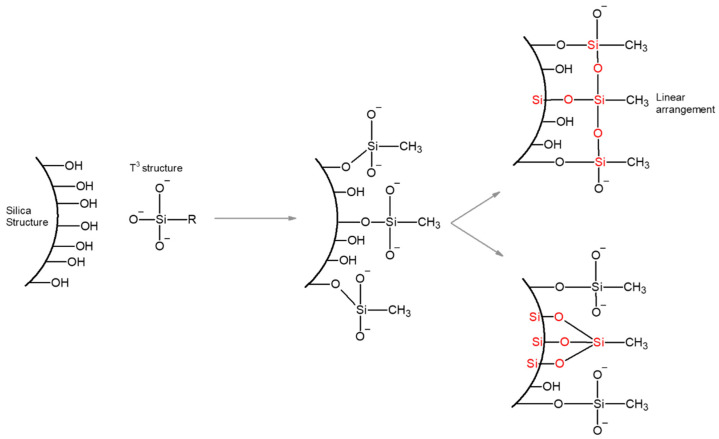
Schematic representation of the linear arrangement of the T^3^ structure [46].

According to the ^29^Si-MAS-NMR results, the Q^n^ region was entirely dominated by the Q^4^ (four BOs and zero NBO) groups showing a high degree of polymerization. The siloxane linkage resulting from the TEOS polymerization underwent a different degree of crosslinking and ended up with both Q^3^ (three BOs and one NBO) and Q^4^ structures only in samples MT-50 and MT-25. The Q^3^ structures in these samples showed the presence of the –OH groups in the incompletely condensed silanes. The samples with extra silylation displayed distinct T^3^ bands only, indicating the high degree of trifunctional silane condensation. The formation of a tridental anchoring of trifunctional silanes by three silanols, which are coexisting on the silica surface and sufficiently close to each other, was very unlikely. These distinct T^3^ structures were possibly caused by the formation of three Si–O–Si bonds as a result of the condensation between the methoxy groups from two neighboring MTMS [46]. Figure 4 represents a schematic representation of a linearized tridentate anchoring.

The prominent T^3^ structures observed in the NMR spectra for all samples accorded with the obvious Si–C bonds obtained in the FTIR spectrum. By contrast, small T^1^ signals, which can indicate the existence of hydroxyl-rich groups on the silica surface, existed in the non-silylated samples in addition to the T^3^ band. The presence of these OH-linked silica sites was also in a good agreement with the FTIR data. In contrast to the silylated samples, all non-silylated samples possessed an additional band that appeared as an incipient shoulder at ~960 cm^−1^ in Figure 1. This indicates the abundance of the Si–O and Si–OH groups retained on the xerogel surface and reveals that performing extra silylation is effective in eliminating the surface silanols and favors material methylation. Aside from the other xerogels, a distinct signal near −15 ppm confirmed the existence of the trimetylsilyl (TMS) groups (–Si (CH_3_)_3_) on the silica skeleton as a result of the modification with TMCS in samples MT-50-S and MT-25-S. In these samples, the number of surface radicals introduced into the network by co-condensation was probably not high enough to cover all the silanol groups on the silica surface because of the decreasing amount of MTMS in the sol. Some open hydroxyl groups were still observed before the modification.

These open hydroxyl groups were successfully replaced with surface radicals by the extra silylation with TMCS. The majority of the terminal groups were replaced with the trimethylsilyl groups through the ≡Si–O–Si(CH_3_)_3_ bonds during the surface modification. The Q^3^ and T^1^ structures in the non-silylated xerogels were converted into the Q^4^ and T^3^ bands, respectively, in the silylated ones.

### 2.2. Morphological Characterizations

#### 2.2.1. N_2_ Sorption Studies

The N_2_ sorption studies were performed to understand the textural properties of the produced silica xerogels in detail. Table 1 depicts the apparent surface area, average pore size, and total pore volumes of the modified and unmodified silica xerogels obtained from the BET and BJH analyses. The porosity and the experimental bulk density of the samples were calculated from their mass-to-volume ratio for the samples after drying according to Equation (1). The pore volume and average pore size of the xerogels were also estimated by using Equations (3) and (4).

**Figure 5:**
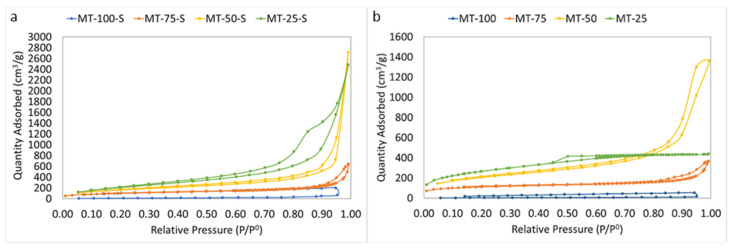
N2 adsorption–desorption isotherms of (**a**) silylated silica xerogels and (**b**) non-silylated silica xerogels.

**Figure 6:**
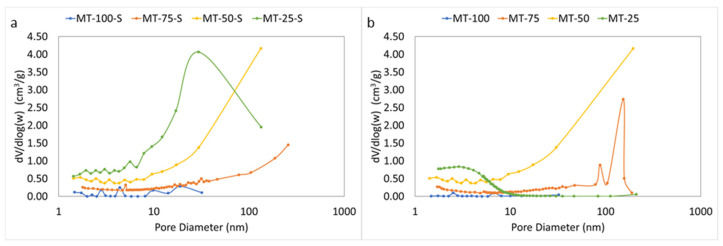
BJH pore size distribution curves of the (**a**) silylated and (**b**) non-silylated silica xerogels.

**Figure 7:**
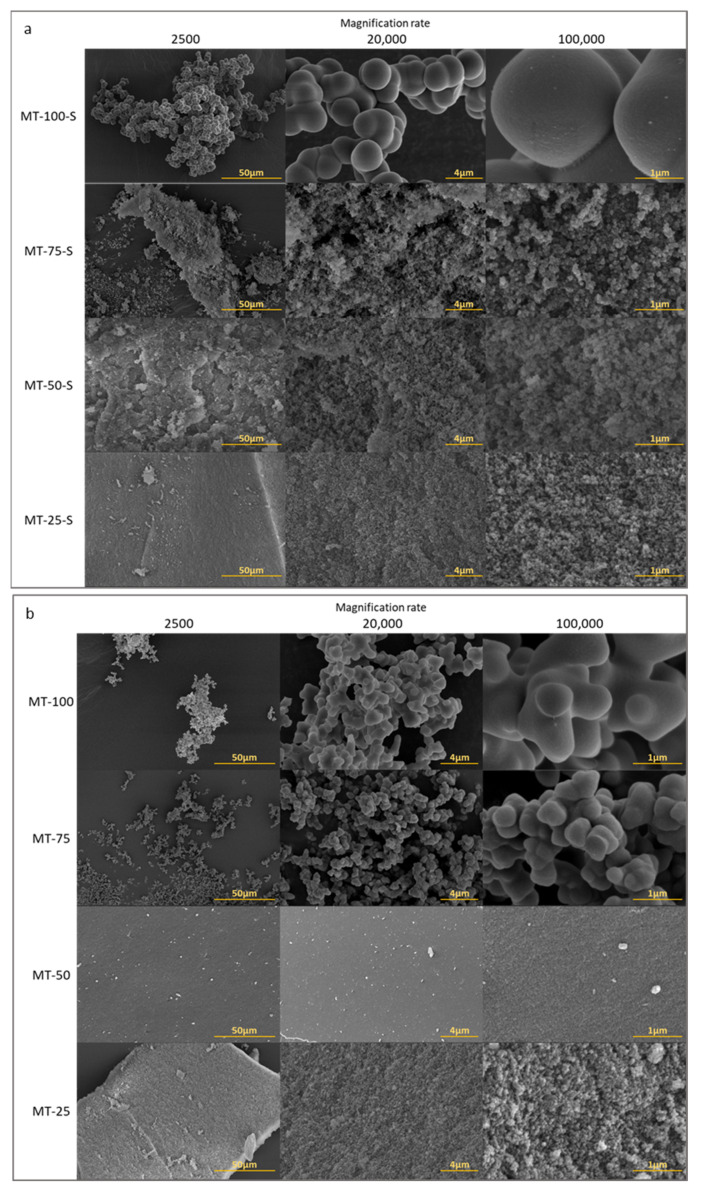
SEM micrographs of (**a**) silylated silica xerogels and (**b**) non-silylated silica xerogels.

Figure 5 and Figure 6 illustrate the N_2_ adsorption–desorption isotherms and pore size distributions of the samples, respectively. Comparing the obtained isotherms with the IUPAC classification, all samples, except MT-100 and MT-100-S, exhibited type IV isotherm, which is a characteristic for mesoporous structures. In both series, all samples, except for MT-25, showed an H3-type of hysteresis indicating a mesoporous network consisting of some macropores not completely filled with pore condensate. For MT-50-S a steep rise in the adsorption branch at high relative pressure also indicates the contribution of macropores in the structure. Unlike other samples, MT-25 exhibited an H4-type hysteresis loop, which is characteristic of narrow slit-shaped pores. The relatively high uptake of the adsorption branch of this sample in low p/p_0_ was related to the micropore filling and an indicator of the existence of obvious micropores along with mesopores in the network. As evident from small average pore diameters (<2 nm), the samples with and without modification (i.e., MT-25 and MT-25-S, respectively) both possessed a serious microporous domain at the lowest MTMS content. At the highest content, the samples MT-100 and MT-100-S displayed a very weak micro/mesoporous structure. The micrometer-sized particle agglomeration and the existence of large voids in these samples, which is visible in the Figure 7, seemed to confirm the nitrogen porosimetry data. Despite the experimentally obtained very low bulk density (<0.096 g/cm^3^) and the high porosity (>95%) of MT-100 and MT-100-S, the mesopore volume and the specific surface area obtained from the BET measurements were quite low and highly different from the rest of the samples. This controversial result is usually attributed to the highly deformable, flexible, macroporous network of these samples which undergoes significant compression during the N_2_ gas intrusion and ultimately results in a substantial underestimation in the experimentally obtained apparent surface area and average pore diameter as indicated in many studies. Husing et.al stated that open hysteresis loop (as seen in Figure 5 for MT-100 and MT-100-S) can also be an indicator for such phenomena especially with a high methyl content [47]. In parallel to their findings, adsorption–desorption isotherms and pore size distribution could not properly be obtained and for the samples MT-100 and MT-100-S. Consequently, serious deviations between the geometrical pore volumes displayed in Table 1 and the pore volumes obtained from the N_2_ sorption experiments are generally attributed to the macropore domination in the material [16,48].

Observations similar to those in this study were also reported by Kanamori et al., concluding that severe phase separation occurs in systems containing only trimetyhlalkoxysilanes as the sole precursor, those that lack a surfactant, or those with an insufficient amount of surfactant. In these systems, the disturbance of the colloidal silica aggregation by phase separation becomes weaker because the extent of phase separation far exceeds that of colloidal aggregation. These systems also strictly indicate a macroporous morphology [49].

The porous texture of all xerogels was also verified in the quantitative BET results. All samples, except for MT-100 and MT-100-S, possessed a high apparent surface area (ASA) ranging from 300 to almost 1000 m^2^/g and a high total pore volume. The obtained ASAs significantly varied with the amount of MTMS incorporated during gelation. The ASA was at its lowest in the highest MTMS content probably due to the generation of macropores by inducing phase separation. The highest ASA values were obtained as the pore sizes became smaller within the network, as in MT-25 and MT-25-S. The pore size generally increased with the increasing MTMS content. The mean pore diameters were low (<3 nm) at a low MTMS content (25%, 50% by vol.), and sharply increased up to 11 nm when the MTMS content increased to 75%. Although the average pore sizes for MT-100 and MT-100-S seemed extremely low, the **d_p,BET_** did not reflect the accurate **d_p_** due to the restriction of the pore size range used in the BJH model. The value of d_p_ * obtained via Equation (4), on the other hand, uses total pore volume of the samples and considers all ranges of pore sizes in the material. As is evident from the SEM micrographs showing certain macroporous domains, and from the d_p_ * values found from Equation (4), we can easily say that samples MT-100 and MT-100-S surely possessed macropores in their network with pore widths greater than 260 nm. Although pore volumes and average pore diameters showed distinct discrepancies between the two methodologies, the obtained results became much closer in the MT-25 and MT-50 series regardless of the applied silylation. The main reason for this is that these samples comprised mostly mesopores and most of the total pore volume was accompanied by pores below 260 nm, which can be found by the nitrogen adsorption–desorption method.

Figure 6 displays the pore size distribution (PSD) curves obtained from the BJH analysis of the adsorption branch of the samples. The isotherm results were reflected well by the BJH outcomes. In the figure, although the pore sizes were mostly located in the mesoporous range in the majority of the samples, the pore distribution still displayed clear variations. At the lowest MTMS content, the pore distribution in the MT-25 tended to shift toward the micropore domain due to the existence of micropores in its structure. Nevertheless, the silylation-applied counterpart of this sample in the other series (MT-25-S) had a broader PSD range. MT-50 and MT-50-S illustrated a wider pore range mostly distributed within the mesoporous range with a small macropore contribution. The PSD of MT-75-S also exhibited a trend similar to MT-50-S. In the non-silylated MT-75, however, a sharp maxima centered above 100 nm was observed confirming the presence of distinct macropores in the network. Although MT-100 and MT-100-S showed some mesopores at certain regions, their proportions were excessively small compared to those of samples with both MTMS and TEOS as the silica precursors and as indicated earlier these samples are mostly comprised of macropores.

#### 2.2.2. SEM Micrographs

The SEM micrographs display the diverse morphology of the synthesized xerogels according to the methyl substitution in Figure 7.

At a high MTMS content (i.e., MT-75, MT-100, and MT-100-S), the particle agglomeration was more distinct; the particles were more regularly spherical in shape; and the network was looser with larger pores. In contrast, decreasing the amount of MTMS led to finer structures with a more compact morphology. Although the size and the cluster radii of the primary particles remained nearly the same with the addition of trifunctional organosilane to the xerogel network, the size of the secondary silica particles significantly varied with the methyl content because the reaction kinetics of classical tetraalkoxysilanes and trialkoxysilanes were not similar. The particles were primarily formed by the hydrolysis and condensation of tetraalkoxysilane (TEOS) and the hydrolysis and polycondensation of trialkoxysilanes occurred on the surface of these silica particles [50]. As the amount of MTMS increased in the sol, larger secondary silica particles were irregularly stacked on each other and exhibited a non-homogenous clustered morphology with randomly distributed pores. At a moderate MTMS content, (i.e., MT-50 and MT-50-S), the sample displayed a more compact morphology with densely packed nanoparticles and nanopores. Xerogels with a more uniform aggregation of the colloidal silica particles were obtained at the lowest MTMS content (i.e., MT-25 and MT-25-S). The porous network formation also became distinct.

In the course of synthesizing inorganic–organic hybrid aerogels, in which trifunctional silanes were incorporated as the silica precursor or co-precursor in the sol–gel system, two phenomena, cyclization and phase separation, are the critical issues that needed to be precisely controlled to adjust the macroscopic morphology in the final material [51]. Cyclization occurs when organosilanes form cyclic species (e.g., polyhedral oligomeric silsesquioxanes) during the sol–gel transition. This species do not take part in the structure because of their low siloxane formation ability and can seriously hamper the gel network homogeneity [52]. Trialkoxysilanes possess a relatively strong phase separation tendency in the water–alcohol solvent compared to that in a system solely comprised of tetraalkoxysilanes [47,49,53]. The sol–gel systems accompanied by phase separation tend to exhibit a spinodal decomposition and the coarsened, bi-continuous network is formed as a result of the spinodal decomposition (as seen in MT-75, MT-100, and MT-100-S).

The increased trialkoxysilane concentration in the sol–gel reactions increases the degree of binding of the hydrophobic radicals to organosiloxane-based oligomers during the sol–gel transition, which negatively affects the compatibility of the silica colloids toward polar solvents and eventually induces a phase separation. The cationic surfactant (CTAB) utilized at a fixed concentration, in this study, helped to turn the hygroscopic nature of these silica oligomers from hydrophobic to more hydrophilic enough to prevent macroscopic phase separation in the sol mixture.

From the micrographs, it is understood that by simply adjusting the methyl content, or in other words, depending on the surfactant/trialkoxysilane precursor ratio and the phase separation tendency, the macro/mesoporosity of the xerogels can be tailored, and this was in very good accordance with the nitrogen sorption measurements. On the other hand, for the samples with highest methyl content (MT-100 and MT-100-S), the increasing quantity of the methyl functionalization by means of silylation caused a slight increase in bulk density and decrease in porosity. Increasing of the system density was also proven by the FT-IR spectra (increasing of the shoulder intensity at around 1180 cm^−1^).

When the methyl content decreased, a transition in the xerogel texture from hierarchical macro/mesoporous to a dominantly mesoporous structure was easily detected and evidenced by the N_2_ porosimetry results. At the low MTMS contents (25 and 50%), the cationic surfactant was enough to completely suppress the spinodal decomposition (or phase separation of the trialkoxysilane). The mesopores were dominantly generated as a result of silica particle aggregation. In the non-silylated samples with a low MTMS content, with the aid of open silanol groups in the network, the colloids were connected to each other via new siloxane bonds on the surfaces. As a result of the increased polycondensation, the connectivity between the secondary silica particles, and hence the network compactness, increased compared to that of the silylated counterparts. Compared to the non-silylated samples, performing extra silylation on MT-25-S, MT-50-S, and MT-75-S minimized the interchain bonding by the attachment of extra non-polar methyl radicals to the siloxane (Si–O–Si) backbone. Silylation decreased the crosslinking degree and the network compactness and enhanced the porous morphology of the samples (Figure 7). The higher total pore volumes obtained from the N_2_ porosimetry (Table 1) also confirmed these results.

At high MTMS contents, the same amount of surfactant could not completely suppress the macroscopic phase separation and the mesh eventually resulted in a hierarchically porous morphology, in which the possible micro/mesopores were embedded within a macroscopic skeleton (MT-75, MT-100, MT-100-S).

### 2.3. Physical Properties

**Figure 8:**
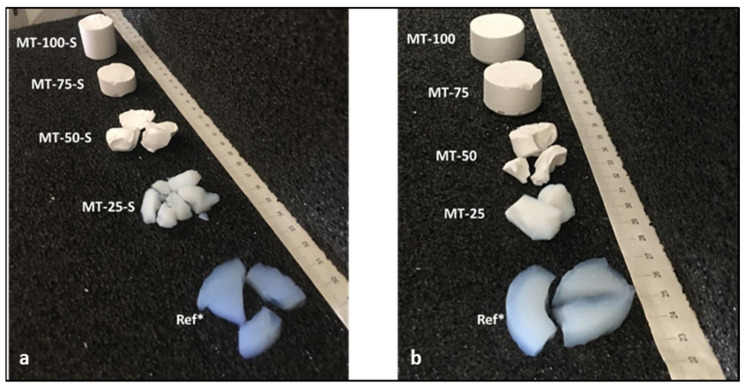
Physical appearances of the (**a**) silylated and (**b**) non-silylated silica xerogels (Ref*: neat aerogel).

The physical appearances and obtained physical properties of the methylated xerogels after APD were displayed in Figure 8 and Table 1, respectively.

During the solvent evaporation from the wet gel pores in the APD, lateral compressive stress occurs on the pore walls to compensate for the fluid loss in the pores. In the absence of radical groups that can prevent undesired surface condensation reactions, the terminal silanol groups on the silica surface converged with each other during the solvent evaporation, consequently forming new siloxane bonds that eventually resulted in an irreversible volumetric contraction of the gel. The number of open silanol groups, and thus the probability of polymerization during condensation, have a direct relation to the type and amount of precursors used in the sol–gel reactions. Therefore, the extent of polymerization in xerogels was controlled herein by varying the MTMS concentration in the sol and conducting extra silylation to acquire monolithic xerogels with a well-defined porous network.

As depicted in the Figure 8, in the samples comprising 25 or 50% of MTMS by volume, an extensive condensation led to a high degree of cross-linking that resulted in a more rigid structure. As the structure became finer and the pore size became smaller in the samples with a low MTMS content (MT-25, MT-50, MT-25-S, and MT-50-S) the capillary pressure exerted on the pore walls of the silica network during solvent evaporation increased severely according to following relation (Equation (5)):

(1) $$P_{r}=-2\gamma_{LV}/r_{m}$$

where $\gamma_{LV}$ is the pore liquid surface tension, and $r_{m}$ is the mean pore radius. With the decreasing pore sizes, the solid skeleton underwent a structural collapse and the samples were obtained only in a granular form with higher bulk densities (Table 1).

Despite the application of the surface modification to wet gels of MT-25-S and MT-50-S prior to the drying step for eliminating the open silanol groups, the samples containing low MTMS still exhibited a poor ability in withstanding capillary pressure during the aging period. Hence, fractures were formed during drying and the sample was obtained as large granules.

Meanwhile, in the samples containing MTMS as a single or major precursor, the number of open silanol groups and the sites available for the condensation reactions that may cause shrinkage during the drying were significantly reduced (i.e., MT-75, MT-100, MT-75-S, and MT-100-S). The clustered structures and the existence of large pores also played a predominant role in alleviating the capillary stress that occurred during solvent evaporation and correlated well with the retention of almost the initial volume of the gels by after the drying stage. Therefore, with the existence of abundant functional methyl groups on the surface and the hierarchically porous structures, these samples maintained their structural integrity as a result of the induced “spring-back effect” after drying and possessed a relatively flexible and monolithic form with very low densities.

### 2.4. Surface Characterizations

**Figure 9:**
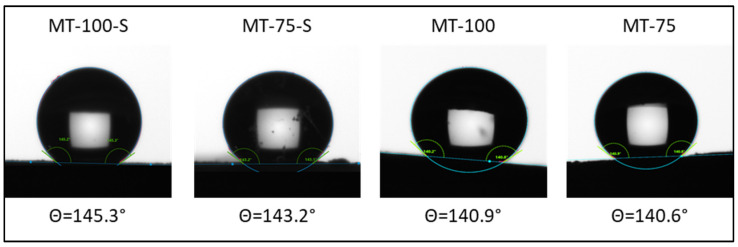
Water contact angles of the silica xerogels.

Hydrophobicity of the monolithic xerogels were revealed via the contact angle analysis and the results are depicted in Figure 9.

The measurements showed that all samples exhibited a hydrophobic behavior with contact angle values greater than 140° due to their high methyl contents. Surprisingly, only slight differences were observed in the obtained contact angles between the samples synthesized with silylation and those that were not silylated. The existence of surface silanols on the non-silylated samples was apparent in the NMR results, but this did not deteriorate the hydrophobicity of the samples to a great extent probably due to their hierarchical porous network. As explained by the Wenzel and Cassie–Baxter roughness-related wettability models, for a hydrophobic surface, the increasing surface roughness can make the material more hydrophobic [54]. The obtained hierarchical meso/macroporous network probably induced the surface roughness of MT-100 and MT-75 and has allowed them to retain their hydrophobic character. Regarding the low-content MTMS samples, although the abundance of in situ-added surface radicals seemed to decrease in the MT-25 and MT-50 (silylated/unsilylated), they still exhibited a hydrophobic character. This finding may confirm the fact that silylation is an effective tool in ensuring the hydrophobic structures regardless of the pre-methylation degree of the wet gels. Although the contact angle measurements could not be performed for the materials having fragmented surfaces (MT-25, MT-25-S, MT-50, and MT-50-S), the hydrophobic character of those samples were visually proven by simply soaking the materials in the water and observing their high water-repelling behavior. A video showing the interaction of the samples with water can be found in the Video S1 in the Supplementary Material.

## 3. Conclusions

This study was performed to analyze the degree of methyl substitution of silica-based xerogels synthesized via the co-gelation approach in achieving a monolithic structure, and to understand the synergetic impacts of co-gelation and ex-situ surface silylation methods on the surface and structural properties of these silica xerogels. For this purpose, a trifunctional organosilane methyltrimethoxysilane (MTMS) was utilized as the silica co-precursor along with the traditionally used tetraethylorthosilicate (TEOS). To investigate the changes in the materials’ morphology and surface characteristics with the methyl substitution, the molar ratio of MTMS/TEOS was varied, and extra silylation with a monofunctional silane (trimethylchlorosilane (TMCS)) was also studied as a second parameter. The results revealed that silylation directly affects the enhancement of the methylation of the silica surfaces both confirmed by the FTIR and ^29^Si-MAS-NMR analyses. The SEM micrographs showed that adjusting the MTMS content led to the phase separation tendency and the simplified tuning of the macroporous structure of the material. The N_2_ sorption analysis proved that the xerogel texture changed from a micro/mesoporous nature to a hierarchical macro/mesoporous nature when the methyl content increased. In the samples containing MTMS as a single or major precursor, the crosslinking degree and the network compactness were significantly reduced (i.e., MT-75, MT-100, MT-75-S, and MT-100-S) and the samples were obtained in a monolithic form with very low densities. Wettability studies also supported the surface methylation with high contact angle values (>140°). The high hydrophobicity, light weight, and monolithic form of these four samples can make these materials practical candidates for potential environmental and biomedical applications in the future.

## 4. Materials and Methods

### 4.1. Materials

In this study, methyltrimethoxysilane (MTMS, 98%) and tetraethylorthosilicate (TEOS, 99%) were selected as the silica precursors. Ethanol, n-hexane, and deionized water were used as the solvents; cetyltrimethylammonium bromide (CTAB, >98%) served as a cationic surfactant; Trimethylchlorosilane (TMCS) was chosen as the monofunctional silylating agent; and HCI and NH_4_OH were selected as the acid and base catalysts. All chemicals were acquired as analytical grade quality from either Sigma Aldrich (Darmstadt, Germany) or Merck Millipore (Darmstadt, Germany) and utilized without further purification.

### 4.2. Methods

The synthesis of the methyl-substituted silica xerogels was performed by following a single step sol–gel method. During the sol preparation, a liquid mixture containing ethanol, water, 0.2 M HCI, and 0.1 g CTAB was first mixed in a beaker for 15 min. The volumetric ratio of EtOH/H_2_O/HCI was kept constant as 1/14/0.4. In another beaker, silica precursors with a gradually increased methyl content by volume as 0%, 25%, 50%, 75%, and 100% (MTMS:TEOS) were mixed with each other and stirred for 10 min. The mixture was then added to an acidic solution and the volumetric ratio of the silica precursors to the total solvents was settled as 1/3. The obtained sol was hydrolyzed for 40 min by vigorous stirring under ambient conditions. After sufficient hydrolysis, the pH value of the sol mixtures was set to 7 by adding 1 M NH_4_OH dropwise to promote the condensation reactions. After 2 mins of mixing, the colloidal solution was taken into cylindrical polypropylene molds for gelation. A complete gelation of all samples was observed within 30 min and the obtained gels were kept at room temperature for 24 h for aging. Following this, the samples were then further aged in fresh ethanol at 25 °C for 24 h to increase their mechanical strength. For better drying, ethanol in the alcogel pores was replaced with n-hexane to decrease the surface tension of the pore fluid for a better drying. The solvent exchange was performed at 25 °C for 12 h.

To investigate the effect of silylation on the surface and pore characteristics of the obtained xerogels, while the first set of the samples remained unmodified, the second set of samples was subjected to a surface modification for 24 h by using 10% TMCS by vol. in n-hexane at 25 °C under an ambient pressure. Regarding the methyl content by volume (MT-Vol. Ratio) and the applied silylation (S), the first set of the samples was abbreviated as MT-0, MT-25, MT-50, MT-75, and MT-100 and the second set was abbreviated as MT-0-S, MT-25-S, MT-50-S, MT-75-S, and MT-100-S.

Prior to drying, all samples were washed with n-hexane twice at 25 °C for 24 h to remove any impurities. Finally, ambient pressure drying was performed at 70 °C overnight and subsequently at 90 °C and 110 °C for 2 h to obtain the methylated silica xerogels.

### 4.3. Characterizations

#### 4.3.1. Chemical Characterization

The entire chemical composition and the methyl substitution of the materials were qualitatively analyzed by FTIR spectroscopy (PERKIN ELMER, Spectrum 100, ABD) in the 600–4000 cm^−1^ wavenumber range.

^29^Si-MAS-NMR analysis was performed on a Bruker Superconducting FT, NMR Spectrometer Avance TM spectrometer at a resonance frequency of 300 MHz to acquire an element specific, potentially quantitative, and more detailed insight on the surface chemistry of the obtained xerogels. The ^29^Si-NMR spectra were collected with a magic angle spinning (MAS) spectrometer with an 8500 Hz spinning rate and a 4 mm probe at 20 °C.

#### 4.3.2. Morphological Characterizations

The bulk density (ρ_b_) was calculated by measuring the dimensions and the mass of the monolithic xerogels. The porosity of the samples was then determined via the following Equation (1) where $\rho_{s}$ represents the density of the solid skeleton. Differing from silica aerogels produced with pure TEOS, for the aerogels made of organically modified silica as in this study, the skeletal density can be taken as 1.43 g/cm^3^; whereas for the materials synthesized only with MTMS, the corresponding skeletal density should be 1.9 g/cm^3^ according to the literature [55]. The percentage linear shrinkage of the materials was determined by using the diameter of the monolithic xerogels before ($d_{0})$ and after (d), the drying stage, via Equation (2):

(2) Porosity (%)=(1−ρbρs)×100

(3) % of linear shrinkage=(1−dd0)×100

The N_2_ sorption studies were conducted in a Micrometrics 3 Flex model pore analyzer to determine the textural properties of the synthesized silica xerogels. Before the analysis, the degassing of the xerogels was carried out at 200 °C overnight, and the adsorption–desorption isotherms were obtained at 77 K. The apparent surface area of the samples was calculated by the Brunauer–Emmett–Teller (BET) method whereas the mesopore volume, pore size distribution, and average pore diameter of the samples were only determined for the size range of 1.4 nm–260 nm according to the Barrett–Joyner–Halenda (BJH) method. In order to define the whole network, with the foresight that there may be macropores in the structure, total pore volume ($V_{p}{}^{*}),$ depending on the bulk density, and average pore size, depending on the total pore volume ($d_{p}{}^{*}),$ were also estimated for the monolithic samples using Equations (3) and (4) [5,55]:

(4) Vp*=1ρb−1ρs

(5) dp*=4(Vp*)SBET

The surface morphology of the samples was characterized by SEM. The analysis was performed on a Carl Zeiss 300VP instrument. The samples were coated with 10 nm of Pd-Au before the analysis to minimize the possible charging effects. The micrographs were obtained at a 20 kV accelerating voltage in a low vacuum mode.

#### 4.3.3. Surface Characterization

The contact angle measurements were conducted via the sessile drop technique by using the Attension Theata Optical Tensiometer combined with a high-speed camera to quantify the degree of hydrophobicity and the surface free energies of the obtained monolithic xerogels. The contact angles were measured from inside and outside of the material and the mean values were taken after five measurements.

## Figures and Tables

**Table 1:** The physicochemical properties of the silica xerogels.

Sample ID	Surface Area, m^2^/g	Ave. Pore Diameter, nm		Total Pore Volume, cm^3^/g		Experimental Bulk Density, g/cm^3^	Porosity, %	% of Linear Shrinkage
	S_BET_	d_p,BET_	d_p_ *	V_p,BET_	V_p_ *	ρ_b_		
MT-100-S	39	1.4	1014	0.12	9.89	0.096	95	2.86
MT-75-S	302	11	100	0.99	7.57	0.121	92	3.57
MT-50-S	613	1.7	34	4.25	5.32	0.166	88	-
MT-25-S	844	1.4	24	4.05	5.06	0.173	87	-
MT-100	11	2.5	5318	0.03	14.63	0.066	97	6.07
MT-75	382	5.8	82	0.57	7.85	0.117	92	7.14
MT-50	729	2.8	20	2.11	3.74	0.225	84	-
MT-25	948	1.4	10	0.67	2.42	0.320	78	-

* the properties are calculated depending on the experimental ρ_b_ and ρ_s._

## Data Availability

The data presented in this study are available on request from the corresponding author.

## Supplementary Materials

The following are available online at https://www.mdpi.com/article/10.3390/gels9010033/s1, Video S1: Hydrophobic interactions of MT-50 and MT-50-S with water.

## References

[1] Hayase G., Kanamori K., Fukuchi M., Kaji H., Nakanishi K. (2013). Facile Synthesis of Marshmallow-like Macroporous Gels Usable under Harsh Conditions for the Separation of Oil and Water. Angew. Chem.-Int. Ed..

[2] Sert Çok S., Koç F., Gïzlï N. (2021). Lightweight and Highly Hydrophobic Silica Aerogels Dried in Ambient Pressure for an Efficient Oil/Organic Solvent Adsorption. J. Hazard. Mater..

[3] Sert Çok S., Gizli N. (2022). Microstructural Properties and Heat Transfer Characteristics of In-Situ Modified Silica Aerogels Prepared with Different Organosilanes. Int. J. Heat Mass Transf..

[4] Mahadik D.B., Lee Y.K., Chavan N.K., Mahadik S.A., Park H.H. (2016). Monolithic and Shrinkage-Free Hydrophobic Silica Aerogels via New Rapid Supercritical Extraction Process. J. Supercrit. Fluids.

[5] Torres R.B., Vareda J.P., Lamy-Mendes A., Durães L. (2019). Effect of Different Silylation Agents on the Properties of Ambient Pressure Dried and Supercritically Dried Vinyl-Modified Silica Aerogels. J. Supercrit. Fluids.

[6] Hasanpour M., Hatami M. (2020). Application of Three Dimensional Porous Aerogels as Adsorbent for Removal of Heavy Metal Ions from Water/Wastewater: A Review Study. Adv. Colloid Interface Sci..

[7] Menaa B., Herrero M., Rives V., Lavrenko M., Eggers D.K. (2008). Favourable Influence of Hydrophobic Surfaces on Protein Structure in Porous Organically-Modified Silica Glasses. Biomaterials.

[8] Kortesuo P., Ahola M., Kangas M., Leino T., Laakso S., Vuorilehto L., Yli-Urpo A., Kiesvaara J., Marvola M. (2001). Alkyl-Substituted Silica Gel as a Carrier in the Controlled Release of Dexmedetomidine. J. Control. Release.

[9] Huang X., Liu Z., Xia W., Zou R., Han R.P.S. (2015). Alkylated Phase Change Composites for Thermal Energy Storage Based on Surface-Modified Silica Aerogels. J. Mater. Chem. A.

[10] Smirnova I., Suttiruengwong S., Arlt W. (2004). Feasibility Study of Hydrophilic and Hydrophobic Silica Aerogels as Drug Delivery Systems. J. Non Cryst. Solids.

[11] Len A., Paladini G., Románszki L., Putz A.M., Almásy L., László K., Bálint S., Krajnc A., Kriechbaum M., Kuncser A. (2021). Physicochemical Characterization and Drug Release Properties of Methyl-Substituted Silica Xerogels Made Using Sol–Gel Process. Int. J. Mol. Sci..

[12] Smith D.M., Stein D., Anderson J.M., Ackerman W. (1995). Preparation of Low-Density Xerogels at Ambient Pressure. J. Non Cryst. Solids.

[13] Bhagat S.D., Rao A.V. (2006). Surface Chemical Modification of TEOS Based Silica Aerogels Synthesized by Two Step (Acid-Base) Sol-Gel Process. Appl. Surf. Sci..

[14] Rao A.P., Rao A.V., Pajonk G.M. (2007). Hydrophobic and Physical Properties of the Ambient Pressure Dried Silica Aerogels with Sodium Silicate Precursor Using Various Surface Modification Agents. Appl. Surf. Sci..

[15] Kanamori K., Aizawa M., Nakanishi K., Hanada T. (2008). Elastic Organic-Inorganic Hybrid Aerogels and Xerogels. J. Sol-Gel Sci. Technol..

[16] Bhagat S.D., Oh C.S., Kim Y.H., Ahn Y.S., Yeo J.G. (2007). Methyltrimethoxysilane Based Monolithic Silica Aerogels via Ambient Pressure Drying. Microporous Mesoporous Mater..

[17] Çok S.S., Gizli N. (2020). Hydrophobic Silica Aerogels Synthesized in Ambient Conditions by Preserving the Pore Structure via Two-Step Silylation. Ceram. Int..

[18] Dudás Z., Len A., Ianăși C., Paladini G. (2020). Structural Modifications Caused by the Increasing MTES Amount in Hybrid MTES/TEOS-Based Silica Xerogels. Mater. Charact..

[19] Lee K.J., Lee J.M., Nam K.S., Hwang H. (2021). Thermal Gelation for Synthesis of Surface-Modified Silica Aerogel Powders. Gels.

[20] Yun S., Luo H., Gao Y. (2014). Superhydrophobic Silica Aerogel Microspheres from Methyltrimethoxysilane: Rapid Synthesis via Ambient Pressure Drying and Excellent Absorption Properties. RSC Adv..

[21] Rao A.V., Pajonk G.M., Bhagat S.D., Barboux P. (2004). Comparative Studies on the Surface Chemical Modification of Silica Aerogels Based on Various Organosilane Compounds of the Type R NSiX4-N. J. Non Cryst. Solids.

[22] Mahadik S.A., Pedraza F., Parale V.G., Park H.H. (2016). Organically Modified Silica Aerogel with Different Functional Silylating Agents and Effect on Their Physico-Chemical Properties. J. Non Cryst. Solids.

[23] Shewale P.M., Rao A.V., Rao A.P. (2008). Effect of Different Trimethyl Silylating Agents on the Hydrophobic and Physical Properties of Silica Aerogels. Appl. Surf. Sci..

[24] Rao A.V., Kulkarni M.M., Amalnerkar D.P., Seth T. (2003). Superhydrophobic Silica Aerogels Based on Methyltrimethoxysilane Precursor. J. Non Cryst. Solids.

[25] Xu B., Cai J.Y., Xie Z., Wang L., Burgar I., Finn N., Cai Z., Wong L. (2012). An Improved Method for Preparing Monolithic Aerogels Based on Methyltrimethoxysilane at Ambient Pressure Part II: Microstructure and Performance of the Aerogels. Microporous Mesoporous Mater..

[26] Lei C., Hu Z., Zhang Y., Yang H., Li J., Hu S. (2018). Tailoring Structural and Physical Properties of Polymethylsilsesquioxane Aerogels by Adjusting NH3·H2O Concentration. Microporous Mesoporous Mater..

[27] Venkateswara Rao A., Bhagat S.D., Hirashima H., Pajonk G.M. (2006). Synthesis of Flexible Silica Aerogels Using Methyltrimethoxysilane (MTMS) Precursor. J. Colloid Interface Sci..

[28] Yang Z., Yu H., Li X., Ding H., Ji H. (2019). Hyperelastic and Hydrophobic Silica Aerogels with Enhanced Compressive Strength by Using VTES/MTMS as Precursors. J. Non Cryst. Solids.

[29] Yue X., Chen J., Li H., Xiao Z., Yu X., Xiang J. (2020). One Pot Rapid Synthesis of Ultra High Strength Hydrophobic Bulk Silica Aerogels. Mater. Chem. Front..

[30] Luo Y., Li Z., Zhang W., Yan H., Wang Y., Li M., Liu Q. (2019). Rapid Synthesis and Characterization of Ambient Pressure Dried Monolithic Silica Aerogels in Ethanol/Water Co-Solvent System. J. Non Cryst. Solids.

[31] Xu B., Cai J.Y., Finn N., Cai Z. (2012). An Improved Method for Preparing Monolithic Aerogels Based on Methyltrimethoxysilane at Ambient Pressure Part I: Process Development and Macrostructures of the Aerogels. Microporous Mesoporous Mater..

[32] Li T., Du A., Zhang T., Ding W., Liu M., Shen J., Zhang Z., Zhou B. (2018). Efficient Preparation of Crack-Free, Low-Density and Transparent Polymethylsilsesquioxane Aerogels: Via Ambient Pressure Drying and Surface Modification. RSC Adv..

[33] Yan X., Hu X., Komarneni S. (2014). Facile Synthesis of Mesoporous MOF/Silica Composites. RSC Adv..

[34] Zu G., Kanamori K., Wang X., Nakanishi K., Shen J. (2020). Superelastic Triple-Network Polyorganosiloxane-Based Aerogels as Transparent Thermal Superinsulators and Efficient Separators. Chem. Mater..

[35] Ding J., Zhong K., Liu S., Wu X., Shen X., Cui S., Chen X. (2020). Flexible and Super Hydrophobic Polymethylsilsesquioxane Based Silica Aerogel for Organic Solvent Adsorption via Ambient Pressure Drying Technique. Powder Technol..

[36] Wu X., Zhong K., Ding J., Shen X., Cui S., Zhong Y., Ma J., Chen X. (2020). Facile Synthesis of Flexible and Hydrophobic Polymethylsilsesquioxane Based Silica Aerogel via the Co-Precursor Method and Ambient Pressure Drying Technique. J. Non Cryst. Solids.

[37] He S., Chen X. (2017). Flexible Silica Aerogel Based on Methyltrimethoxysilane with Improved Mechanical Property. J. Non Cryst. Solids.

[38] Gopal N.O., Narasimhulu K.V., Rao J.L. (2004). EPR, Optical, Infrared and Raman Spectral Studies of Actinolite Mineral. Spectrochim. Acta-Part A Mol. Biomol. Spectrosc..

[39] Cruz-Quesada G., Espinal-Viguri M., Garrido J. (2022). Novel Silica Hybrid Xerogels Prepared by Co-Condensation of TEOS and ClPhTEOS: A Chemical and Morphological Study. Gels.

[40] Cruz-Quesada G., Espinal-Viguri M., López-Ramón M.V., Garrido J.J. (2021). Hybrid Xerogels: Study of the Sol-Gel Process and Local Structure by Vibrational Spectroscopy. Polymers.

[41] Das G., Mariotto G., Quaranta A. (2006). Microstructural Evolution of Thermally Treated Low-Dielectric Constant SiOC:H Films Prepared by PECVD. J. Electrochem. Soc..

[42] Stojanovic A., Paz Comesaña S., Rentsch D., Koebel M.M., Malfait W.J. (2019). Ambient Pressure Drying of Silica Aerogels after Hydrophobization with Mono-, Di- and Tri-Functional Silanes and Mixtures Thereof. Microporous Mesoporous Mater..

[43] Li J., Cao J., Huo L., He X. (2012). One-Step Synthesis of Hydrophobic Silica Aerogel via in Situ Surface Modification. Mater. Lett..

[44] Bauer F., Freyer A., Ernst H., Gläsel H.J., Mehnert R. (2001). Application of Temperature-Programmed Oxidation, Multinuclear MAS NMR and DRIFT Spectroscopy to the Surface Characterization of Modified Silica Nanoparticles. Appl. Surf. Sci..

[45] Li Z., Zhao S., Koebel M.M., Malfait W.J. (2020). Silica Aerogels with Tailored Chemical Functionality. Mater. Des..

[46] Bauer F., Gläsel H.J., Decker U., Ernst H., Freyer A., Hartmann E., Sauerland V., Mehnert R. (2003). Trialkoxysilane Grafting onto Nanoparticles for the Preparation of Clear Coat Polyacrylate Systems with Excellent Scratch Performance. Prog. Org. Coat..

[47] Ehgartner C.R., Grandl S., Feinle A., Hüsing N. (2017). Flexible Organofunctional Aerogels. Dalt. Trans..

[48] Scherer G.W., Smith D.M., Stein D. (1995). Deformation of Aerogels during Characterization. J. Non Cryst. Solids.

[49] Kanamori K., Kodera Y., Hayase G., Nakanishi K., Hanada T. (2011). Transition from Transparent Aerogels to Hierarchically Porous Monoliths in Polymethylsilsesquioxane Sol-Gel System. J. Colloid Interface Sci..

[50] Venkateswara Rao A., Haranath D. (1999). Effect of Methyltrimethoxysilane as a Synthesis Component on the Hydrophobicity and Some Physical Properties of Silica Aerogels. Microporous Mesoporous Mater..

[51] Shimizu T., Kanamori K., Nakanishi K. (2017). Silicone-Based Organic–Inorganic Hybrid Aerogels and Xerogels. Chem.-A Eur. J..

[52] Kanamori K., Nakanishi K. (2011). Controlled Pore Formation in Organotrialkoxysilane-Derived Hybrids: From Aerogels to Hierarchically Porous Monoliths. Chem. Soc. Rev..

[53] Kanamori K., Nakanishi K., Hanada T. (2009). Spinodal Decomposition in Siloxane Sol-Gel Systems in Macroporous Media. Soft Matter.

[54] Khanmohammadi Chenab K., Sohrabi B., Rahmanzadeh A. (2019). Superhydrophobicity: Advanced Biological and Biomedical Applications. Biomater. Sci..

[55] Lamy-Mendes A., Girão A.V., Silva R.F., Durães L. (2019). Polysilsesquioxane-Based Silica Aerogel Monoliths with Embedded CNTs. Microporous Mesoporous Mater..

